# Area Deprivation Index as a Surrogate of Resilience in Aging and Dementia

**DOI:** 10.3389/fpsyg.2022.930415

**Published:** 2022-06-29

**Authors:** Maria Vassilaki, Ronald C. Petersen, Prashanthi Vemuri

**Affiliations:** ^1^Department of Quantitative Health Sciences, Mayo Clinic, Rochester, MN, United States; ^2^Department of Neurology, Mayo Clinic, Rochester, MN, United States; ^3^Department of Radiology, Mayo Clinic, Rochester, MN, United States

**Keywords:** area deprivation index, resilience, socioeconomic, cognitive impairment, dementia

## Abstract

Area deprivation index (ADI), a tool used to capture the multidimensional neighborhood socioeconomic disadvantage across populations, is highly relevant to the field of aging and Alzheimer’s disease and Alzheimer’s disease related dementias (AD/ADRD). ADI is specifically relevant in the context of resilience, a broad term used to explain why some older adults have better cognitive outcomes than others. The goal of this mini-review is three-fold: (1) to summarize the current literature on ADI and its link to cognitive impairment outcomes; (2) suggest possible mechanisms through which ADI may have an impact on AD/ADRD outcomes, and (3) discuss important considerations when studying relations between ADI and cognitive as well as brain health. Though difficult to separate both the upstream factors that emerge from high (worse) ADI and all the mechanisms at play, ADI is an attractive proxy of resilience that captures multifactorial contributors to the risk of dementia. In addition, a life-course approach to studying ADI may allow us to capture resilience, which is a process developed over the lifespan. It might be easier to build, preserve or improve resilience in an environment that facilitates instead of hindering physical, social, and cognitively beneficial activities. Neighborhood disadvantage can adversely impact cognitive impairment risk but be at the same time a modifiable risk factor, amenable to policy changes that can affect communities.

## Introduction

Chronological age is the strongest risk factor for dementia, and dementia prevalence is expected to rise due to the aging of the population, reaching potentially close to 150 million cases worldwide by 2050 (Livingston et al., 2017), with most of the new cases of dementia occurring in the low and middle-income countries (LMICs) (Ashby-Mitchell et al., 2020). Alzheimer’s disease and Alzheimer’s disease related dementias (AD/ADRD) have devastating consequences not only for the individual but have a major impact on the family, society, and the health care economies (Petersen, 2018; Stephan et al., 2018; Tochel et al., 2019). Thus, interventions that target modifiable AD/ADRD risk factors are of utmost importance.

Persons from historically underrepresented groups and socially disadvantaged populations are disproportionately affected by AD/ADRD (Powell et al., 2020). Research is still limited about the association between neighborhood socioeconomic deprivation with cognitive impairment (Marengoni et al., 2011; Yaffe et al., 2013; Kind and Buckingham, 2018; McCann et al., 2018). However, conditions adversely associated with a person’s risk for cognitive impairment (e.g., higher rates of cardiovascular diseases, diabetes, multimorbidity, stress levels, health behaviors) (Roberts and Knopman, 2013; Roberts et al., 2015; Resende et al., 2019) are also associated with living in socioeconomically deprived neighborhoods (Kind and Buckingham, 2018; McCann et al., 2018; Chamberlain et al., 2020).

Area level deprivation measures, like the area deprivation index (ADI) (Kind and Buckingham, 2018), encompass geographic area-based estimates of the socioeconomic disadvantage of neighborhoods. These composite measures integrate indicators for several social determinants of health (Powell et al., 2020), such as education, employment, housing, and poverty (Kind and Buckingham, 2018; McCann et al., 2018), and allow us to study how living in socioeconomically disadvantaged neighborhoods may adversely affect health and disease outcomes (Kind and Buckingham, 2018; McCann et al., 2018; Chamberlain et al., 2020).

The concept of resilience has been used to explain better cognitive outcomes in a subset of individuals in the context of aging and dementia and is based on complex, interactive mechanisms (Stern et al., 2020) involving a person’s demographics, genetics, and exposures over the lifespan. Therefore, cognitive impairment (in part due to low resilience) could also be affected by neighborhood socioeconomic disadvantage, as socioeconomically deprived neighborhoods experience more difficult living, working, and learning conditions (Zuelsdorff et al., 2020) and adverse impact on their health and health behaviors.

In this work, we will review literature on ADI as a measure of socioeconomic deprivation of neighborhoods and expand on the potential mechanisms through which ADI could be associated with resilience in aging and dementia. We hypothesize that ADI, reflective of comorbidities and lifestyles at present and through the lifespan, would be a useful quantifiable measure of resilience reflective of cognitive impairment risk due to socioeconomic status differences. We use resilience here in the context of better-than-expected cognitive performance (Arenaza-Urquijo and Vemuri, 2020).

## Area Deprivation Index

The effect of multiple individual measures of socioeconomic status (e.g., education, income, occupation) on health has been studied more in the past but recently, more attention is focused on the effect of neighborhood context on health (Chamberlain et al., 2022). The ADI is a composite measure of neighborhood socioeconomic disadvantage at the Census Block Group level, -the closest approximation to a “neighborhood” - using 17 census measures including education, employment, income, poverty, and housing characteristics (Singh, 2003; Kind et al., 2014). ADI is publicly available, and values can be downloaded from the Neighborhood Atlas^®^ website^1^, the University of Wisconsin, School of Medicine and Public Health (Kind et al., 2014; Kind and Buckingham, 2018). Briefly, the ADI values are provided in national percentile rankings at the block group level (i.e., a block group with a ranking of 1 shows the lowest level of neighborhood disadvantage within the nation, but a ranking of 100 suggests the highest level of neighborhood disadvantage). The ADI values are also provided in deciles created by ranking the ADI within each state (a block group ranking of 1 shows the lowest level of neighborhood disadvantage within the state, and 10 specifies the highest ADI (most disadvantaged) within the state).

## Area Deprivation Index as a Surrogate of Lifestyle and Morbidity

Health risk behaviors (e.g., smoking, drinking, being sedentary) and health-promoting behaviors (e.g., physical exercise, interpersonal interaction, spiritual growth, stress management) constitute one’s lifestyle (Wang and Geng, 2019). In the US, nearly 40% of deaths could be linked to lifestyle-related behavioral factors (e.g., tobacco use, poor diet, physical inactivity, alcohol consumption), which are associated with an increased chronic disease burden (including AD/ADRD) but are modifiable (Mokdad et al., 2004; Bauer et al., 2014). Lifestyle might not be entirely a personal choice; it is influenced by various social factors, including socioeconomic status, and could even mediate the relationship between socioeconomic status and one’s health (Wang and Geng, 2019).

A recent report estimated that twelve modifiable risk factors (i.e., less education, physical inactivity, low social contact, alcohol consumption, hypertension, hearing impairment, smoking, obesity, depression, diabetes, traumatic brain injury, and air pollution) could account for 40% of dementia cases worldwide (Livingston et al., 2020). Several of these modifiable risk factors are associated also with living in socioeconomically deprived neighborhoods, as aforementioned (Roberts and Knopman, 2013; Roberts et al., 2015; Kind and Buckingham, 2018; McCann et al., 2018; Resende et al., 2019; Chamberlain et al., 2020). Neighborhoods have characteristics that can impact health behaviors, environmental factors related to socioeconomic status (e.g., lead exposure, air pollution), and socioeconomically deprived neighborhoods could provide less opportunities for cognitively beneficial activities (e.g., social, recreational, physical, cognitive activities) (Diez Roux and Mair, 2010; Krell-Roesch et al., 2017, 2019; George et al., 2020; Hunt et al., 2021). The impact of preventive interventions (e.g., addressing these modifiable AD/ADRD risk factors) could be high and potentially even higher for LMICs where more dementia cases occur (Livingston et al., 2020).

Chronic conditions and multimorbidity (e.g., the co-occurrence of ≥2 conditions in a person) are more prevalent in persons with lower socioeconomic status (Rawshani et al., 2016; Pathirana and Jackson, 2018; Chamberlain et al., 2022). Living in socioeconomically deprived neighborhoods adversely affects not only health (e.g., higher rates of cardiovascular diseases, diabetes, stress levels, premature mortality, worse all-cause, and cardiovascular mortality), but also health behaviors, access to food, safety, and education, (Kind and Buckingham, 2018; McCann et al., 2018; Chamberlain et al., 2020) beyond also the effects of individual measures of socioeconomic status (Ludwig et al., 2011; Rawshani et al., 2016). As many of these conditions are associated with mild cognitive impairment (MCI) and dementia risk, area-level socioeconomic deprivation could contribute to late-life cognitive impairment (Roberts and Knopman, 2013; Roberts et al., 2015; Resende et al., 2019).

Area deprivation index was associated with multimorbidity in a cohort of nearly 200,000 people even after adjusting for education (an individual-level socioeconomic variable). This association was stronger in younger ages and women (Chamberlain et al., 2020). In addition, the risk of most chronic conditions (e.g., hypertension, congestive heart failure, coronary artery disease, cardiac arrhythmias, hyperlipidemia, stroke, diabetes, dementia, depression, schizophrenia, substance abuse disorders, and anxiety) increased with increasing ADI (Chamberlain et al., 2022). Patterns of associations were in general, similar for men and women, but the associations were modestly stronger in women for some of the chronic conditions (hyperlipidemia, diabetes, cardiac arrhythmias, coronary artery disease, arthritis, osteoporosis, and depression) (Chamberlain et al., 2022). However, not all studies point to such conclusions, as previous reports suggest that the socioeconomic gradient is steeper for men than women for health outcomes, except possibly heart disease (Deguen et al., 2010; Phillips and Hamberg, 2015).

Area deprivation index is estimated independent of sex and gender, it is a composite measure capturing education, employment, income, poverty, and housing characteristics at the census block group level, as aforementioned. However, sex and gender disparities and neighborhood disadvantage disparities need to be considered while studying resilience in AD/ADRD. The sex- and gender-specific changes in the balance between resilience and pathogenesis risk factors vary over the AD/ADRD disease course; however, the cause of such sex and gender differences is not clearly understood (Mielke et al., 2022).

**FIGURE 1:**
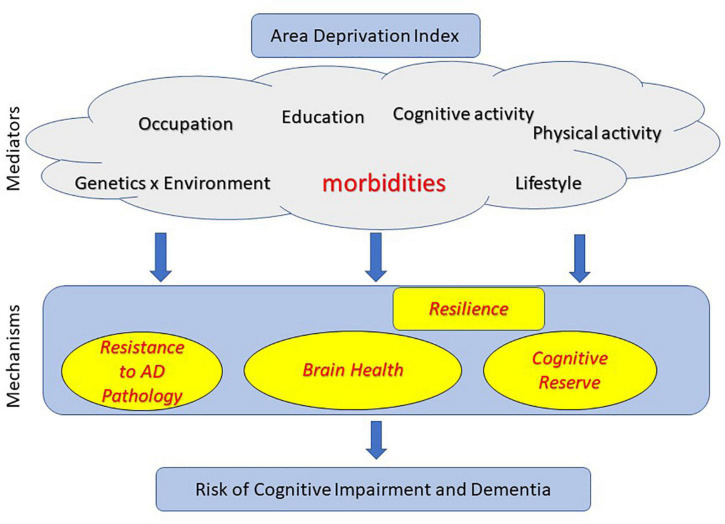
Mechanisms through which area deprivation index (ADI) may influence risk of cognitive impairment, Alzheimer’s disease and Alzheimer’s disease related dementias (AD/ADRD).

In summary, ADI as a variable is reflective of a multitude of factors as illustrated in Figure 1. While associations may vary in different populations, ADI may be reflective of lifestyle broadly and much more closely associated to comorbidities.

## Mechanisms Through Which Area Deprivation Index May Influence Cognitive, Alzheimer’s Disease and Alzheimer’s Disease Related Dementias Outcomes

There has been tremendous research in the field of protective and risk factors that influence cognitive outcomes in aging and dementia. There is increasing understanding that risk of cognitive impairment is explained by multiple pathways along the life course. In this section, we discuss the three possible mechanistic pathways through which ADI may be associated with cognitive and AD/ADRD outcomes – (i) lower AD pathology [also termed as “Resistance” (Arenaza-Urquijo and Vemuri, 2018) where lower than expected AD pathology is observed]; (ii) better brain health (Stern et al., 2018); (iii) higher cognitive reserve (Stern et al., 2018). We discuss the latter two pathways in the context of “Resilience” to AD pathologies wherein some individuals cope with pathologies better than others. Here we highlight literature where mediators (identified in Figure 1) have been shown to impact risk of cognitive impairment through each of these pathways.

### Factors Contributing to Lower Alzheimer’s Disease Pathological Burden

“Resistance to AD pathologies” has been suggested through a multitude of protective factors. While sleep has been the most consistently shown protective factor against amyloidosis through the clearance of neurotoxic waste (Spira et al., 2013; Xie et al., 2013; Carvalho et al., 2018); gene x environment interactions are also increasingly being recognized as contributors to Resistance to AD (Wirth et al., 2014; Coomans et al., 2022). Though physical and cognitive lifestyle has been proposed to influence AD pathological burden (Landau et al., 2012; Okonkwo et al., 2014), these findings have not been consistent across studies. Because ADI reflects several of these mediator factors, one would expect a relationship between ADI and AD neuropathology. A recent study found evidence for this association by suggesting that living in the most disadvantaged neighborhood decile was associated with more than twice the odds of Alzheimer’s disease neuropathology (i.e., diffuse plaques or neuritic plaques) (Powell et al., 2020).

### Factors Contributing to Better Brain Health

There are a greater number of factors that have been shown to have an impact on brain health (which reflects overall brain structure and function and commonly measured using MRI and FDG-PET). Comorbidities, that could be more prevalent in persons with higher neighborhood socioeconomic deprivation or high ADI, have been found consistently to be associated with greater neurodegeneration independent of amyloidosis (Vassilaki et al., 2016; Vemuri et al., 2017). Even in midlife before the onset of neurodegenerative pathologies, poor general health status was associated with worse brain health (Neth et al., 2019). Overall general health is greatly influenced by several upstream processes such as cognitive activity, physical activity, lifestyle, education, and occupation. Several of these mediating factors have a bidirectional relationship with neighborhood disadvantage. A review provided evidence that socioeconomic status (SES) is associated with developmental trajectories of gray matter structure (Rakesh and Whittle, 2021). Hunt et al. (2020) found that higher socioeconomic disadvantage, as measured by ADI, was associated with lower hippocampal and total brain tissue volume, lending support for this pathway from ADI to risk of cognitive impairment.

### Factors Contributing to Higher Cognitive Reserve

Cognitive Reserve refers to the property of the brain that allows for cognitive performance that is better than expected given the degree of life-course related brain changes and brain injury (Definition from the https://reserveandresilience.com/). Therefore, in addition to protective pathways through lower AD pathological burden and better brain health, lower ADI (or higher neighborhood SES) will act through the cognitive reserve pathway to reduce the risk of cognitive impairment.

Cognitive reserve is influenced by genetic and environmental exposures throughout the lifespan, which would be impacted by neighborhood socioeconomic deprivation. For example, in Apolipoprotein E ε4 carriers, years of schooling were associated with significantly delayed cognitive endpoints in patients with late-onset AD, possibly suggesting the neuroprotective effects of educational activities and their association with cognitive reserve (de Oliveira et al., 2018). We hypothesize that a rich in resources environment with opportunities for leisure and physical activities, community centers for social interactions, public libraries, and safety would promote cognitive reserve. Neighborhood SES is reflective of factors that could affect development (e.g., quality of education, access to parks and libraries or health care, crime, and pollution) (Rakesh and Whittle, 2021). An important proxy of cognitive reserve in the literature so far has been education levels, usually measured at the individual level, but also reflects access and educational opportunities of the area-level or neighborhood socioeconomic status. Children in poverty are more likely to have developmental delay, worse performance on cognitive and achievement tests than their more fortunate peers and their SES is associated with educational accomplishment, psychological welfare, and health decades later, as reviewed in Johnson et al. (2016). Socioeconomic difficulties, education in preschool years, in childhood and adolescence, and financial resources have been associated with both cognitive development and cognitive impairment in the life course (Cha et al., 2021). This evidence supports the downstream effects of the SES and ADI throughout life on cognitive reserve. In fact, in previous studies in LMICs (Mukadam et al., 2019; Ashby-Mitchell et al., 2020) low education and physical inactivity contributed to a greater fraction of dementia cases than depression and diabetes, reflecting the potential for positively impacting cognitive reserve going forward and AD/ADRD postponement and prevention in the countries that most dementia cases occur.

## Important Considerations and Future Directions

Through multiple risk mechanisms (e.g., reduced educational opportunities, or access to quality medical care or healthy food, chronic stress, increased morbidity), neighborhood disadvantage can adversely impact cognitive impairment risk but be at the same time a modifiable risk factor, amenable to policy changes that can affect communities.

Therefore, studying the broad range of factors intertwined with ADI and mechanisms through which ADI may impact cognitive outcomes is crucial. As discussed, socioeconomic conditions across the lifespan could be associated with the risk of cognitive impairment through three main pathways. Though difficult to separate both the upstream factors that emerge from worse ADI and all the mechanisms at play, ADI is very attractive as a proxy of resilience that captures multifactorial contributors to the risk of dementia. Here are some open challenges and research avenues on the horizon along with new opportunities.

Area deprivation index (Zuelsdorff et al., 2020) is a validated composite measure of neighborhood disadvantage, funded by the National Institutes of Health and publicly available for the United States and Puerto Rico through the Neighborhood Atlas^®^ (Kind and Buckingham, 2018). Thus, the research community can easily incorporate ADI in their studies and assess disparities in brain resilience and area-level socioeconomic deprivation. As suggested by the National Institute on Aging Health Disparities Research Framework, the socioeconomically disadvantaged populations are included in the priority populations for health disparities in aging research (Hill et al., 2015; National Institute on Aging, 2022). In addition, the Neighborhood Atlas^®^ provides ADI ranking within a state and nationally, allowing further comparisons between studies within a state or nationwide.

The life course approach suggests that health is influenced by past exposures even decades earlier (Jones et al., 2019). Resilience in dementia and aging is a process developed over the lifespan, while lifetime exposures interact and accumulate, resulting in chronic diseases (Lynch and Smith, 2005; Arenaza-Urquijo and Vemuri, 2020). However, few studies have recreated the area-level socioeconomic deprivation in the lifespan to examine its association with cognitive impairment (George et al., 2020). A life-course approach could allow the study of SES exposures during gestation and the different life epochs (i.e., childhood, young adulthood, midlife, older adulthood), that accrue and interact over the years to modify resilience and cognitive impairment risk (Kuh et al., 2003; George et al., 2020). Such studies would assist in the identification of Resilience area-level SES markers across the lifespan and possibly specific most vulnerable life epochs, which is also important in understanding mechanisms of action.

Changes in cognition associated with neighborhood characteristics (e.g., available resources like proximity to public transport or community centers) appear to be lesser than changes related to one’s health behaviors; however, beneficial changes in neighborhood deprivation could be easier to implement than changing an individual’s health behaviors in a deprived area (Clarke et al., 2015). Thus similarly, it might be easier to build, preserve or improve brain resilience in an environment that facilitates instead of hindering physical, social, and cognitively beneficial activities. This is especially important, as postponing dementia onset by even 1 year could result in nine million fewer cases worldwide than predicted by 2050 (Brookmeyer et al., 2007).

## Author Contributions

PV conceived the idea. PV and MV equally contributed to drafting the manuscript. RP provided critical review and input. All authors contributed to the article and approved the submitted version.

## Conflict of Interest

MV received research funding from F. Hoffmann-La Roche Ltd and Biogen in the past and consults for F. Hoffmann-La Roche Ltd; she currently receives research funding from NIH/NIA and has equity ownership in Abbott Laboratories, Johnson and Johnson, Medtronic, AbbVie, and Amgen. RP was a consultant for Roche, Inc., Biogen, Inc., Merck, Inc., Eisai, Inc., Genentech, Inc. and Nestle, Inc.; receives publishing royalties from Mild Cognitive Impairment (Oxford University Press, 2003), UpToDate and receives research support from the National Institute of Health. The remaining author declares that the research was conducted in the absence of any commercial or financial relationships that could be construed as a potential conflict of interest. The handling editor declared a past co-authorship with the authors, PV.

## Publisher’s Note

All claims expressed in this article are solely those of the authors and do not necessarily represent those of their affiliated organizations, or those of the publisher, the editors and the reviewers. Any product that may be evaluated in this article, or claim that may be made by its manufacturer, is not guaranteed or endorsed by the publisher.

## Footnotes

1
https://www.neighborhoodatlas.medicine.wisc.edu/
